# A systematic review of different models of home and community care services for older persons

**DOI:** 10.1186/1472-6963-11-93

**Published:** 2011-05-09

**Authors:** Lee-Fay Low, Melvyn Yap, Henry Brodaty

**Affiliations:** 1 Dementia Collaborative Research Centre, School of Psychiatry, Faculty of Medicine, AGSM Building, University of NSW, Sydney NSW 2052 Australia

## Abstract

**Background:**

Costs and consumer preference have led to a shift from the long-term institutional care of aged older people to home and community based care. The aim of this review is to evaluate the outcomes of case managed, integrated or consumer directed home and community care services for older persons, including those with dementia.

**Methods:**

A systematic review was conducted of non-medical home and community care services for frail older persons. MEDLINE, PsycINFO, CINAHL, AgeLine, Scopus and PubMed were searched from 1994 to May 2009. Two researchers independently reviewed search results.

**Results:**

Thirty five papers were included in this review. Evidence from randomized controlled trials showed that case management improves function and appropriate use of medications, increases use of community services and reduces nursing home admission. Evidence, mostly from non-randomized trials, showed that integrated care increases service use; randomized trials reported that integrated care does not improve clinical outcomes. The lowest quality evidence was for consumer directed care which appears to increase satisfaction with care and community service use but has little effect on clinical outcomes. Studies were heterogeneous in methodology and results were not consistent.

**Conclusions:**

The outcomes of each model of care differ and correspond to the model's focus. Combining key elements of all three models may maximize outcomes.

## Background

Across the world, the proportion of older persons is projected to grow from 6.9% of the population in 2000 to a 19.3% in 2050 [1]. This expanding aged population has resulted in an increasing need for long-term care services for the frail aged. Costs and consumer preference have led to a shift from the long-term institutional care of aged older people to home and community based care [2,3], a pattern that is anticipated to grow.

Home and community care services (otherwise known as domiciliary, non-medical home care or social care) aim to assist the older persons to live independently in their homes, and to maintain or enhance their quality-of-life for as long as possible. A range of services may contribute to this aim including home nursing, house cleaning, home maintenance, shopping, transport, day care, social outings, home visits and allied health (podiatry, physiotherapy, etc). Services are delivered through a range of sectors including public health (national, state, county or district), social services, and private for profit or not-for-profit organizations. The funding and administrative systems through which services are delivered differ across and within countries.

A common criticism of home and community services is that they are fragmented, resulting in poor outcomes and wasted resources [4,5]. Multiple services offered by different providers to increasingly disabled older persons with multiple needs often compromise coordination. This criticism led to the introduction of case management (also known as care management or case coordination). This has been defined as a collaborative process of assessment, planning, facilitation and advocacy for options and services to meet an individual's health needs, through communication and coordination of available resources, to promote quality cost effective outcomes [6]. Reviews have suggested that community based case management has clinical benefits for persons with severe mental illness [7] and diabetes [8]. Systematic reviews have reported that case management improves outcomes for frail elderly persons and reduces health care utilization [9,10].

However, demonstration programs showed that case management does not necessarily produce coordinated care, as health and social service systems may not allow case managers to have control over the supply or availability of services [11]. As a result, integrated care has become a major theme of healthcare reform in some regions and countries [12]. Integrated care has been defined as a discrete set of techniques and organizational models designed to create connectivity, alignment and collaboration within and between the cure and care sectors at the funding, administrative and/or provider levels [13]. The level of integration can differ - an integrated system could have linkages between sectors, or explicit structures to coordinate care across sectors, or be fully integrated such that resources are pooled from multiple sectors to be used most efficiently and effectively [14]. There have been suggestions that integration may improve partnership processes rather than impact on services and care recipient outcomes [15]. A review focusing on the features of integrated systems for older persons found that some integrated systems could improve outcomes, satisfaction and/or costs [12].

Recently, consumers have been advocating for consumer directed care, where consumers and their caregivers make choices regarding the care they receive [16]. The amount of consumer choice ranges from selecting the type of services or selecting the service provider to hiring and supervising care staff, and from selecting how care credits are spent to being given the cash to purchase services. Consumer directed care is conceptualized as giving consumers greater awareness, control and responsibility for their health care spending, and therefore incentive to consider both cost and quality when making healthcare decisions [17]. Consumer directed care has been criticized as potentially shifting costs towards the consumer, raising barriers to needed care, and hampering consumer choice by limited information and system restraints [18]. Consumer-directed home care has been trialed in several countries including the Netherlands, England, Germany, France, USA and Austria [19,20]. In Austria, consumer directed care is the only choice as the traditional model of agency directed care is not available. Most of the evaluations in these countries focused on satisfaction rather than functional outcomes or quality of care. We identified no reviews focusing on the health outcomes of consumer-directed care.

The aim of this review is to evaluate the outcomes of case management, integrated care and consumer directed home and community care services for older persons, including those with dementia.

## Methods

Literature searches were performed in MEDLINE, PsycINFO, CINAHL, AgeLine, Scopus, and PubMed using the key phrases ("community care" or "home care" or "community nursing" or "day care" or "respite care" or " case management" or "integrated care" or "consumer directed care") and ("ageing" or "aging" or "aged" or "older" or "elderly" or "dementia" or "Alzheimer$") from 1994 to May 2009. Key phrases were entered in the title, abstract and keywords fields unless this option was not available in which case all fields were searched.

Abstracts were reviewed and articles that met following criteria were included:

1) Written in English.

2) Evaluating the delivery of case managed, integrated or consumer directed home and community services using quantitative outcomes (see below for definitions). Home and community services could include but could not be limited exclusively to medical care.

3) The sample was community dwelling, with either a majority aged 65 years and over, or with a subsample of persons aged 65 and over for whom results were reported separately.

4) The sample was not selected because they had a specific medical illness, except for dementia.

**Figure 1:**
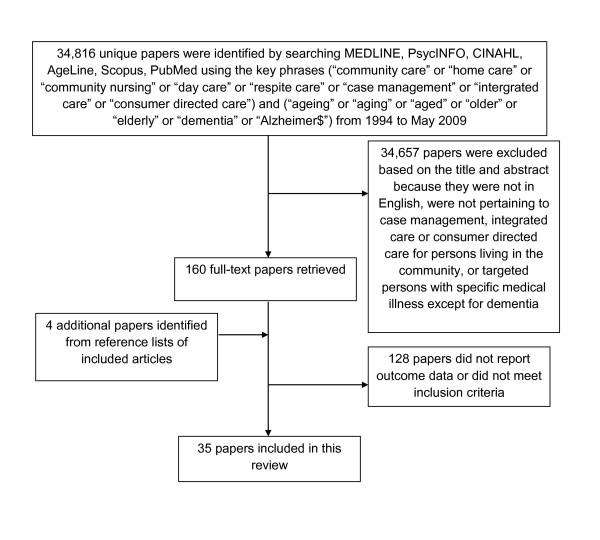
**Article selection process**.

The search yielded 34,816 unique articles. Two authors independently read the titles and abstracts and excluded ineligible papers (see Figure 1). After this exclusion process, 163 full text articles were obtained and reviewed and 35 papers were finally included in the study.

For each included study, methodological quality was rated on a Scale for Rating Quality of Studies [21]. This was modified by eliminating the item on use of standardized diagnostic criteria as no medical condition was required for study inclusion. The maximum total score possible on this scale was 15 points. Differences on quality ratings were resolved through discussion. Methodological quality was used as a measure of the value of the evidence presented, however no studies were excluded based on quality.

Information on study design, demographics, recruitment methodology, intervention description, outcomes and key results were extracted from the studies by one author and checked by a second. Full text was also retrieved for relevant review articles. Articles were grouped by the model of community care being evaluated. Case management was defined as interventions where a central worker provided assessment, care planning, coordination of services and ongoing follow-up. Integrated care was defined as interventions where the services were coordinated at a system level rather than focusing on individual consumers. Consumer directed care was defined as interventions where consumers were explicitly given choice and/or control of services.

Where possible, effect sizes were estimated and described. Cohen's d (d = (_1_-_2_)/SD) was used as the effect size measure of differences between two groups. Effect sizes were defined based on published recommendations as small (d ≤ 0.2 or OR/HR ≥1.3 or OR/HR ≤ 0.77), medium (d ≤ 0.5 or OR/HR ≥1.5 or OR/HR ≤ 0.68) or large (d ≥0.8 or OR/HR ≥2.0 or OR/HR ≤ 0.5) [22,23].

## Results

A summary of the results of outcomes reported in two or more papers for any model of care (case management, integrated care and consumer directed care) is presented in Table 1. This table reports the results by model of care with each letter in the table representing one study, and indicating the study design and effect size where known. Cells in the table with a greater number of letters indicate greater evidence, particularly when the letters indicate that the studies are randomized controlled trials (R).

**Table 1:** Summary of outcomes reported in two or more studies for different models of care for intervention participants relative to controls

	Case management			Integrated care			Consumer directed care		
	Higher	No difference	Lower	Higher	No difference	Lower	Higher	No difference	Lower
**Clinical outcomes (ideally increased)**									
Function (ADLs/IADLs)	R^+++^, N, N	R, N		O	R, R, N		O	N	
Cognition	R^+++^	N, N, O			R, N			N	
Medication management	R, O^++^	N						N	
Quality of life	R	R		N				R, N	
Physical health	O^+^				R		O	R, N	
Social interaction or support			O^+^		R				
**Clinical outcomes (ideally decreased)**									
Depression, psychological health		R, N	R^+++^		R, R, N, O		R	N	
Risk of mortality		R, R, O	R^+++^		R, R, O				N
Caregiver burden/distress		R, O			R			N	
Pain			N		R, O				
**Satisfaction (ideally increased)**									
Satisfaction with care		R		R^+++^	R, O		O, O, O		
Caregiver satisfaction	O^+++^			R	O				
Life satisfaction	R^++^						O		
**Service use**									
Risk of nursing home admission		R	R^++^, R^++^, R^+^, R^+^	O^+^	R, R, N				
Risk of hospital admissions		R	R^+++^, R^+^	N, O^++^, O	R,R				
Risk of emergency admissions	R^+^	R, O	R^++^	N, O	R				
Community service use	R^+++^, R^++^			R^++^, O			R, O		
Length of hospital stay		R, O	R^+++^	N	O				

R = Randomized Controlled Trial, N = Non-randomized Controlled Trial, O = Observational study (case controlled, cross-sectional, longitudinal or retrospective); ^+ ^= small, ^++ ^= medium, ^+++ ^= large effect size; effect sizes were reported whenever possible.

### Case management (see Table 2)

**Table 2:** Case management

Author (year)	Study name/Location; Study design; Intervention Length	Participant group; n (% female); Age ( ± SD)	Study groups	Outcomes and Results	Quality Rating
Gagnon (1999)	Quebec, Canada RCT 10 months	≥70 years who had visited an emergency department in the previous year n = 427 (58.1% F) = 81.6	Participants were assigned nurse case managers who operated using the Promotion of Autonomy Framework. Case managers created and implemented a care plan and coordinated the work of all healthcare and service providers involved in care. There were approximately 28 recorded telephone contacts and 36 home visits per person. For controls, hospital and community services were provided separately.	Over 10-months, participants in the intervention group were readmitted to the emergency department significantly more frequently than controls (p = 0.041, d = 0.2). No significant differences were found between the two groups on quality of life, satisfaction with care, functional status, admission to hospital, and length of hospital stay from baseline to 10 months.	14
Vickrey (2006)	Dementia care quality intervention trial California, USA Cluster RCT 1 year	≥65 years with dementia receiving Medicare with an informal caregiver n = 408 (54.9% F) = 80.1 ± 6.6	Case managers trained to use care management software and provided with a care plan manual conducted assessments and 6 monthly reassessments of participants, designed and implemented care plans in collaboration with caregivers, taught skills and provided ongoing follow-up. Seminars were held for primary care providers at participating health care organisations. Controls received usual care.	After 18 months the proportion of guidelines adhered to was significnatly higher in intervention (64%) compared to controls (24%; p ≤ 0.001). Intervention participants had higher rates of receiving information or services from ≥1 community agency (RR = 1.5, 95% CI 1.0-1.9), respite care (p ≤ 0.03), home health aide services (p ≤ 0.03), professional carer services (p ≤ 0.03), enrollment in a wandering program (p = 0.001), cholinesterase inhibitor use (p = 0.032), health related quality of life (p = 0.034) and health care quality (p ≤ 0.011). Intervention caregivers had higher confidence in caring (p ≤ 0.01), caregiving mastery (p ≤ 0.01), social support (p = 0.029) and met needs for problem behaviours (p ≤ 0.012). There were no differences in caregiver health related quality of life.	14
Alkema (2007), Shannon (2006)	California, USA RCT 1 year	>65 years, enrolled in Medicare health plan, rated as being at risk of future healthcare service use n = 781, 823 (65.3% F) = 83.3	A care manager (care advocate) operating via telephone evaluated needs, made referrals to additional services and called monthly to moitor progress, offer support and coaching, provide additional information and assistance and follow-up to ensure linkages were establised. Controls received usual care including Medicare managed care.	After the 12-month intervention, the case managed care group had lower mortality than controls (OR = 0.45; p = 0.006). However, at 24-month follow-up, mortality differences between the groups were not significant (p = 0.198). After 12 months participants were more likely to use primary care physicians (OR = 2.05, 95% CI 1.28-3.28), were less likely to be admitted to hospital (OR = 0.43, 95% CI 0.22-0.84) and had fewer hospital days (OR = 0.39, 95% CI 0.17-0.86) compared to controls. There were no differences between groups on emergency department and specialist use.	13
Bernabei (1998)	Rovereto, Italy RCT 1 year	≥65 years n = 199 (70.9% F) = 81.0 ± 7.3	Participants received case management and care planning from a community geriatric evaluation unit and general practitioners. Case managers conducted assessments every 2 months, monitored the provision of services, provided extra help as requested and were available to deal with problems. Controls received usual care including non-case managed community services	Over 1 year the intervention group improved on function (ADLs, p < 0.001, d = 6; IADLs, p < 0.05, d = 3) and depression (p < 0.05, d = 4) and declined less on cognition (p < 0.05, d = 4), compared to the control group. Over 1 year, the intervention group had lower risk of admission to a nursing home (HR = 0.81, 95% CI: 0.57-1.16), acute hospital (HR = 0.74, 95% CI: 0.56-0.97), or emergency (HR = 0.64, 95% CI: 0.48-0.85) compared controls. There were no differences in 1 year mortality.	12
Shapiro (2002)	USA RCT 1 year 6 months	≥60 years on waiting list to receive social services n = 105 (85.7% F) = 77.2	Individualized care plans were developed by a geriatric nurse together with participants and caregivers after a thorough in-home geriatric assessment. Case managers coordinated the delivery of services which were prescribed and changed to address specific needs and problems. Controls received usual care.	After 18 months, participants in the intervention group were less likely to be institutionalized or die than those in the comparison group (combined as a single endpoint, OR = 0.18, p = 0.029). The intervention group had improved on Satisfaction with Social Relationships (F = 2.59, p < 0.05, d = 0.45), Environmental Mastery (F = 3.71, p < 0.01, d = 0.54), and Life Satisfaction (F = 3.18, p < 0.05, d = 0.53). No statistically significant difference was found for depression.	12
Eloniemi-Sulkava (2001)	Finland RCT 2 years	≥65 years with dementia and caregivers n = 100 (53.0% F) = 79.4	A nurse case manager with access to a physician provided advocacy, round the clock comprehensive support, continuous and systematic counseling, annual training courses, follow-up calls, in-home visits and assistance with arrangements for social and healthcare services. The frequency of contacts varied from 5 times a day to once a month. Controls received usual care.	During the first 6 months, the rate of institutionalization was significantly lower in the intervention group than in controls (HR = 0.12, 95% CI: 0.02-0.93) but this benefit decreased over time (HR = 1.18, 95% CI: 1.02-1.36). The estimated probability of staying in community care for 6, 12, and 24 months was 0.98, 0.92, and 0.63 in the intervention group and 0.91, 0.81, and 0.68 in the control group, respectively.	12
Miller (1999), Newcomer (1999a, 1999b), Shelton (2001)	Medicare Alzheimer's Disease Demonstration (MADDE) USA RCT 3 years	Persons diagnosed with dementia enrolled in Medicare A and B n = variable (see results) For Newcomer 1999a: 40% F = 79 ± 8 years	MADDE participants received case management (with a ratio 1:30 for Model A or 1:100 for Model B) and 80% subsidy of service costs (up to $489 for Model A or $799 for model B). Controls received usual care.	After 1 year there was increased use of any home care service (OR = 2.77, 95% CI 2.40 - 3.0) and adult day care (OR = 2.23, 95% CI 1.92-2.60) [n = 5209] Over 3 years there were no differences on nursing home entry rates (n = 8095). After 3 years there were no differences in the change in caregiver burden or depression. [n = 5307]. Over 3 years caregivers in MADDE had a lower likelihood than controls of any hospitalization (OR = 0.58, 95% CI 0.35-0.97), but not of emergency department use, length of hospital stay or number of hospitalizations (n = 412). There were no differences between Model A and Model B in any of the outcomes tested.	12
Kinney (2003)	USA RCT 2 years	Enrollees of Indiana's state case management program and/or the Medicaid home and community-based services waiver program for the aged (≥65 years) and disabled n = 1006 (77.5% F) = 67.7	The intervention involved two computer-assisted methods for individualized care planning. The Normative Treatment Planning (NTP) program assessed needs and prescribed services using a standard set of algorithms. The Client Feedback System (CFS) program provided systematic feedback on participant satisfaction to service providers. Participants were randomly assigned to receive none, one or two of the interventions. The control group had case managers who prepared a non-computerized care plan.	Over 2 years perception of needs met (p < 0.05, d = 0.027) and service satisfaction (p < .05, d = 0.027) improved in the NTP compared to the control group. The CFS group had significantly higher satisfaction than the control group (p < 0.05, d = 2.7) but not greater perception of needs met. There were no statistically significant differences in perception of needs met and satisfaction between the group that used both NTP and CFS and the control group.	11.5
Marek (2006)	USA NRCT 1 year	≥64 years n = 85 (80% F) = 77.1	Participants received nurse care coordination in addition to a local care program, Missouri Care Options (MCO), which included basic and advanced personal care, nurse visits, homemaker care, and respite care. Care coordinators conducted a comprehensive admission assessment, created a care plan and coordinated health and social services. Controls were recruited from a similar neighborhood and received the basic MCO program with limited nursing visits and were more likely to be white.	After 12 months, the intervention group improved significantly more than the control group on pain (OASIS M00420; <0.01), dyspnea (p = 0.03), and function (p = 0.01). No significant differences were found over time between groups in emotional stability, medication management, cognition and incontinence.	9
Morales-Asencio (2008)	Spain NRCT 6 months	Homebound persons requiring assistance for daily activities n = 258 = 76.3	A case manager made home visits, conducted assessments, established links with and coordinated other health institutions and professionals, arranged technical assistance at home, provided education telecare for the participants and education and support for caregivers. The control group received visits according to their health demands and at baseline had fewer functional limitations than participants.	The intervention group had significantly lower scores on activities of daily living function and family function compared to the control group at baseline (p = 0.021; p = 0.023 respectively). These differences no longer occurred at six months (p = 0.222; p = 0.142). Cognitive status and instrumental activities of daily living were lower in the intervention than the controls at both baseline (p = 0.042; p = 0.008) and 6 months (p = 0.008; p = 0.007).	8
Gravelle (2007)	Evercare, England Longitudinal observational study 1 year 9 months	≥65 years n = ~7000 Evercare practices (mean age not reported)	Participants were monitored by advanced practice nurses who developed individualized care plans with the participant, general practitioner and other staff. Control data were gathered from all non-Evercare practices in England. At baseline, intervention practices had significantly higher rates of admission and use of emergency bed days and faster growth rates in admissions for the general population aged ≥65.	Over 21 months, the intervention had no significant effect on rates of emergency admission, emergency bed days, and mortality for the whole Evercare sample or a high risk subsample with a history of two or more emergency admissions in the preceding 13 months in comparison to the control group.	7
Onder (2007)	Aged in Home Care Project (AdHoC), Europe Retrospective observational study 1 year	≥65 years and receiving home care services n = 3292 (73.6% F) = 82.3 ± 7.3	The case management group comprised participants living in Finland, Iceland, Italy, Sweden & the UK. Participants in these countries had case managers who conducted assessments, dealt with problems that arose, monitored the provision of services, worked with geriatric evaluation units to design and implement individualized care plans and who provided additional services as needed. Controls lived in Czech Republic, Denmark, France, Germany, the Netherlands and Norway where case management was not available. Controls were more likely to be women, live alone, be physically active, had more severe cognitive impairment and a lower prevalence of daily pain and a number of chronic diseases and unexpected weight loss than participants.	During the 1-year follow-up, the risk of nursing home admission was significantly lower in the case management group compared to controls (OR = 0.56, 95% CI: 0.43-0.63).	7
Bierlein (2006)	Canada Longitudinal observational 6 months	>65 years, 22% were cognitively impaired n = 179 (65% F) = 80 ± 7.38	Participants were assigned case coordinators and had access to various community health services. There was no control group.	After 6 months, participants' scores improved on the physical (p < 0.001, d = 0.4) and mental health subscales (p < 0.001, d = 0.4) of the SF-8. Risk of institutionalization decreased significantly (p < 0.03, d = 0.1). However there was a statistically significant deterioration on social interaction (p < 0.04, d = 0.2) and instrumental support (p < 0.001, d = 0.3). Subjective support scores (p = 0.88) and cognitive scores (p = 0.68) did not change significantly.	7
Onder (2008)	AdHOC Retrospective observational	≥65 years already receiving home care services n = 4007 (74.1% F) = 82.3 ± 7.3	See Onder, 2007 above	Compared to the control group, more participants in the case management group had blood pressure measured in previous 2 years (OR = 1.31, 95% CI 1.08-1.59), received influenza vaccination in the last 2 years (OR = 2.08, 95% CI: 1.81-2.39) and had medication reviewed in the last 6 months (OR = 1.69, 95% CI: 1.42-2.01). Compared to the control group, caregivers of participants in the intervention group were more likely to be able to continue in caring activities (OR = 0.49, 95% CI: 0.35-0.69) and were less dissatisfied (OR = 0.47, 95% CI: 0.29-0.73). There was no significant difference for caregiver distress (OR = 1.04, 95% CI: 0.78-1.38).	6.5

NRCT = Non-randomized controlled trial; RCT = Randomized controlled trial;

On average, the methodological quality for studies of case management was highest of all the models of home and community care reviewed. There were seven randomized controlled trials (three focusing on persons with dementia), two non-randomized trials and three observational studies with non-matched controls comparing case managed care to usual non-coordinated care [24-37]. One observational study did not include a control group [38], and one randomized trial evaluated the effects of a computerized system in the care management process [39]. Different methods of case management were evaluated such as telephone-based case management [28], computer program assisted case management [40] and case management in combination with cost subsidies [30-32]. There were usually few details about the 'usual care' received by controls in terms of the types and ease of access to services available, however this probably differed by locality.

As shown in Table 1, case management improves function, improves different aspects of medication management, increases use of community services and reduces nursing home admission; however this was not the case for all studies. There were also positive results for other clinical outcomes and decreasing hospital admissions but not consistently across studies. It was difficult to quantify differences in the intensity of case management provided between studies; however studies that reported more positive outcomes did not appear to have provided more intensive case management.

### Integrated care (see Table 3)

**Table 3:** Integrated care

Author (year)	Study name/Location; Study design; Intervention Length	Participant group; n (% female); **Age (****± SD)**	Study groups	Outcomes and Results	Quality Rating
Beéland (2006)	System of Integrated Care for Older Persons (SIPA), Canada RCT 1 year 10 months (= 572 days)	≥65 years n = 1230 (71% F) = 82	Participants received care from multidisciplinary teams who delivered integrated care through the provision of health and social services and coordination of hospital and nursing home care, monitoring protocols and providing mobilized resources, including intensive home care, group homes, and a 24-hour on-call service. Controls received usual home care services including nursing, rehabilitation, physician, personal, and social services with limited time and availability and no case management.	Over 22 months significantly more SIPA participants compared to controls received home health (OR = 1.72 95% CI: 1.20-2.46) and home social care (OR = 2.16, 95% CI: 1.60-2.91). There were no significant differences between the groups in mortality or admissions to emergency, hospital or nursing homes. Caregivers' satisfaction with care after 1 year was significantly higher in the intervention group than the control group. There were no significant differences on participants' satisfaction with care, chronic diseases, depression, cognition, functional limitations, daily function, and caregiver burden between the intervention and the control groups.	12.5
Hammar (2007)	Finland Cluster RCT 6 months	≥65 years without dementia n = 668, 22 municipalities (74.0% F) = 81.7	Participants were assigned a home nurse and home helper who planned and integrated home care services with other service providers and hospital staff. Controls were from municipalities without case management or integration. Controls had a smaller number of diagnoses than participants.	At 3-week follow-up, physical mobility significantly improved in the intervention group (p < 0.002) compared to controls but the effect was lost at 6-month follow-up. At 3-week and 6-month follow-ups, there were no significant changes between the two groups on energy, sleep, pain, emotional reactions, and social isolation. There were no differences in self-rated health, daily function, rates of mortality, institutionalization and hospitalization.	12.5
Fischer (2003)	Kaiser Permanente Northwest, USA Longitudinal observational 5 years	Enrollees of Social Health Maintenance Organization (SHMO) ≥65 years n = 18143 (63.7% F) = 75	Participants enrolled in the SHMO received case management and coordination to integrate the delivery of long-term care within the medical care system. Services included care coordination, home nursing visits, homemaking, transportation, adult day care and nursing home respite. Controls resided in an area where the SHMO was terminated and at baseline were younger and had fewer chronic health conditions and less utilization of acute and nursing home inpatient days compared to participants.	Over 5 years, there was an increased probability of nursing home placement for the control group compared to the intervention group (OR = 1.43, 95% CI: 1.15-1.79, p = 0.002). Over 5 years there was no difference in mortality between the intervention and the control group (OR = 1.02, 95% CI: 0.87-1.20, p = 0.828).	12
Atherly (2004)	Program of All Inclusive Care for the Elderly (PACE), USA Cross-sectional	>55 years n = 265 (mean age not reported)	Participants received care from the PACE interdisciplinary teams whom conducted comprehensive assessments and delivered preventive, primary, rehabilitative, supportive, and end-of-life care integrated into a complete health care plan. PACE also attempted to limit unnecessary hospital and nursing home use. Controls were eligible older persons who declined PACE services.	Participants in the PACE group had higher satisfaction on Perceived Interpersonal Quality (p = 0.0006, d = 0.3) and Decision Making (p < 0.0001, d = 0.2) scales compared to controls. There were no differences on family satisfaction.	8.5
Bird (2007)	Hospital Admission Risk Program; (HARP), Australia NRCT ≥90 days (= 227 ± 104 days)	>55 years n = 316 (51.3% F) = 75.3 ± 8.5	Participants were allocated a care facilitator who linked them to all required acute and community services. They also ensured effective communication and exchange of relevant information between services including specialist medical clinics, allied health therapies and carer support services. Controls were eligible older persons who declined participation. No demographic differences were detected at baseline between controls and participants.	Comparing the 12 months pre-recruitment and post-recruitment, participants in the intervention group had a 20.8% reduction in emergency visits (p < 0.001), 27.9% reduction in hospital admissions (p < 0.001), and 19.2% reduction in bed-days (p < 0.001). In the 12 months pre-recruitment and post-recruitment older persons who declined participation showed a non-significant 5.2% increase in emergency visits, 4.4% reduction in hospital admissions, and 15.3% increase in inpatient bed-days.	8
Kane (2006)	PACE and Wisconsin Partnership Program (WPP), USA Longitudinal Variable length	≥65 years n = 1285 (77.3% F) = 77.8	PACE group as above Participants enrolled in WPP were offered choice of care, setting, and manner in which their service was delivered and were able to keep their primary physician, whereas PACE enrollees were not given these choices. Enrollees in PACE were more likely to be women, older, non-White and eligible for Medicaid only (ie not low-income older persons or disabled).	Per person-month of program enrollment, the PACE group had fewer hospital admissions (OR = 0.682, p < 0.001), preventable hospital admissions (OR = 0.589, p < 0.01), hospital days (p < 0.05), emergency visits (p < 0.001), and preventable emergency visits (p < 0.05) than WPP. There was no significant difference between the two groups in the length of hospital stays.	8
Brown (2002)	UK NRCT 18 months	≥65 received a social services assessment after referral from study general practice N = 393 (67% F) = 81 (65-99)	Intervention participants were assessed and managed by social service departments (SSD) co-located with general practices. SSDs met weekly with general practice staff, largely for cross-referrals. Control participants resided in a county of similar population and size which were managed by traditional SSDs.	There were no differences between rates of mortality and nursing home placement after 18 months. In the intervention group time to assessment was shorter than controls (p = 0.039, d = 0.24), and there was an increase in quality of life over 18 months (p = 0.08) not apparent in controls. There were no differences in changes over 18 months on daily function, mental functioning or depression.	8
Wieland (2000)	PACE, USA Longitudinal Up to 8 years	>55 years n = 5478 (71.1% F) = 78.9 ± 8.9	PACE group as above Data were compared to the general Medicare population of older and disabled Americans.	Time to hospitalization for PACE was 773 days (median; 95% CI: 725-814) comparable to Medicare aged and Medicare disabled populations. Annual short-term bed use in PACE showed a decline and was comparable with the general Medicare population, 2046 (in 1998) versus 2014 (in 1997) respectively (no statistical test performed).	8
Weaver (2008)	All-Inclusive Long-term Care, USA Longitudinal Up to 36 months	Older persons veterans (≥55 years) n = 368 (3.8% F) = 76.1	Three Veterans Affairs (VA) medical centers served as study sites, each providing a different program of care: The VA as sole provider program: VA provided all care including homemaker and home health aides, adult day care and health needs. The VA and PACE partnership program: VA provided hospitalization, short-term nursing home for sub acute rehabilitation, subspecialty consultation, laboratory imaging, and pharmacy services while PACE assumed responsibility for primary care, adult day health care, transportation, home health care, homemaker and other supportive care needs. The VA as care manager program: Contracted for PACE to provide all care, veterans did not use VA healthcare services while enrolled in PACE.	Compared to 6 months before program entry, by program discharge there was a significant increase in adult day health care use in all three models (p < 0.001). In the VA as care manager model, there was a significant increase in home care use (p < 0.001) and nursing home use (p < 0.02), but no such increases were found for the other two models. No statistically significant differences were observed in all models in hospital admissions per patient, total inpatient days per patient, nursing home admissions per patient, nursing home days per patient, inpatient, and outpatient clinic use.	7
Temkin-Greener (2002)	PACE, USA Longitudinal Variable length	>55 years n = 2263 = 80	PACE group as above. Data were compared to the general Medicare population of older and disabled Americans.	The probability of death at home for PACE participants (45.0%) was twice as great as the probability of death at home for the Medicare population of older Americans (no statistical test performed).	7
Kane (2002)	WPP (as above) Case controlled	≥65 years n = 1163 78% F = 78.7	WPP described above. Controls were community options program recipients a Medicaid home and community based waiver program who receive a variety of community services designed to meet their care needs but receive their medical care from fee-for-service Medicare providers matched on age and gender from within the same county (in-area controls) and from non-WPP county (out-of-area controls).	Dependency for daily self-care was lower in WPP than in area and less consistently in out-of-area controls (p ranged from 0.000 to 0.033). Over the previous 3 months fewer WPP received homemaker (p < 0.001), but more WPP received nurse, home delivered meals, special transportation, adult daycare, outpatient rehabilitation and physical therapy than both control groups (p ranged from 0.000 to 0.033). There were no differences between groups on depression, pain and unmet needs, use of medical equipment or informal care. The few differences on the 21 satisfaction items were not consistent across control groups.	7

NRCT = Non-randomized controlled trial; RCT = Randomized controlled trial.

There were two randomized controlled trials and two non-randomized trials of integrated compared to non-integrated care [41-44]. There were seven observational studies, six of which evaluated variants of the Program of All Inclusive Care for the Elderly (PACE) [45-51]. The services received by control groups were not well described in most papers, however most controls appeared to receive non-case managed medical and home care services.

Overall, integrated care did not improve clinical outcomes (see Table 1). Fully integrated care programs (e.g. PACE and the Kaiser Permanente Northwest) were associated with greater use of community and hospital services; however the methodological quality of these studies was relatively low. The higher quality randomized and non-randomized trials evaluated partial integration models where services were formally linked and coordinated, however these were more likely to report significant effects on clinical or service use outcomes. Thus it was difficult to evaluate whether fully integrated programs result in better outcomes than programs where linkages are created between disparate systems.

### Consumer-directed care (see Table 4)

**Table 4:** Consumer-directed care

Author (year)	Study name/Location; Study design; Intervention Length	Participant group; n (% female); **Age (****± SD)**	Intervention; Control	Outcomes and Results	Quality Rating
Meng (2005)	Medicare Primary and Consumer Directed Care Demonstration USA RCT 12 months	≥65 years, enrolled in Medicare A & B, ≥2 ADL or ≥3 IADL limitations and been hospitalized, in residential care or received home health care in last 12 months or ≥2 emergency visits in past 6 months n = 1394 (70% F) = 80 ± 8 years	3 intervention groups: 1. Voucher group could choose how to spend ≤$200 p/month, advised and financially managed by voucher specialist 2. Disease management health promotion nurse taught disease management skills, implemented behaviour change strategies, and facilitated conferences with primary care physicians 3. Combination of 1 and 2 Controls received usual Medicare benefits.	The voucher group increased the probability of using personal assistance services (p = 0.002) as did the combination group (p < 0.001). The combination group also increased the probability of use of skilled home health care (p = 0.03).	10
Wiener (2007)	Washington, USA Cross-sectional	Medicaid beneficiaries receiving home and community services n = 513 (72.9% F) ≥65 years: 55%	Participants in the consumer-directed care group were responsible for hiring, orienting, supervising, and finding replacements for their paid caregivers. Participants in the agency-directed care group included those residing in assisted living and residential aged care.	In subsample of participants ≥65 years, thosereceiving consumer-directed services were more satisfied with paid personal assistance compared to those receiving agency-directed care (p < 0.05).	9
Glendinning (2008)	Individual Budgets Pilot Program UK RCT 6 months	Social service recipients, subsample of persons ≥65 years n = 263 66% F Mean age not given	Intervention participants were assigned an individual budget based on a needs assessment which could be spent on large range of services and equipment including hiring family and relatives. They were assisted by a care coordinator. The 13 sites also attempted with varying success to integrate resources from several funding streams. Controls received standard social care.	At 6 months, there were no significant differences betewen individual budget recipients and controls on quality of life, self or informant-rated health or care needs. Indivdiual budget recipients were significantly more likely to score above the cutoff on a screening tool for psychological morbidity (45%) than controls (29%; p < 0.05).	
Carlson (2006)	Cash and Counseling USA RCT (evaluation only at 9 months)	Medicaid beneficiaries - subsamples aged ≥65 years in Arkansas and New Jersey and ≥60 years in Florida N = 2353 Mean age not given	Intervention group could choose how to spend allowance from broad range of equipment and services including hiring relatives - advised by a consultant (counselor). Control group received Medicaid benefits as usual.	Arkanses and New Jersey intervention participants had significantly higher hours of paid care (p < ≤ 0.001), lower hours of unpaid care (p = 0.036; p = 0.034) and were more satisfied with the way the paid caregiver provided care, with overall care arrangements and way of spending life (all p <.001) than controls. In New Jersey intervention particpants wre more likely to have made an equipment purchase or home or vehicle modification (p = 0.039) and had lower rates of falls (p = 0.009)and development or worsening of contractors (p = 0.002). There were no differences between groups on bedsore development and rates of uninary tract infections. In Florida there were few differences between groups which may be because only 39% had received the allowance by the evaluation.	8
Giannini (2007)	Bologna, Italy NRCT 2 years	Older persons needing help in ≥2 ADLs or severely chronically ill and MMSE <24/30 n = 121 = 83.7 ± 6.4 years	The primary caregiver received vouchers to buy 4 to 24 hours per day of home care attendance from health providers. Controls had recently been discharged from hospital and received usual assistance from Public Health and Social Care Services.	Mortality was lower in the consumer-directed care group than in controls at 6 and 24 months (p < 0.05). At 24 months, there were no statistically significant differences between the changes in the two groups on daily function, cognition, clinical burden of medical conditions, severity of cognitive impairment, behavioral and psychological symptoms, caregiver burden, depression, number of drug used and quality of life.	7/
Benjamin (2000)	USA Cross-sectional	Adults (>18 years) in the California Management and Information Payrolling System n = 1095 (72.9% F) 51.2% were ≥65 years	The consumer-directed group recruited and hired their own providers, and trained, supervised, and replaced them as needed. Participants used up to 283 hours of services per month including personal care, household, paramedical, protective supervision and medical transportation. Participants were placed in the agency-based group and receieved services from home care agencies if judged inappropriate for consumer direction.	Participants in the consumer-directed group reported better outcomes than the agency-based group on sense of security, (p < 0.001), unmet activities of daily living needs (p < 0.05), and service satisfaction (technical quality, p < 0.001; service impact, p < 0.001; general satisfaction, p < 0.001; interpersonal manner, p < 0.001). The groups did not differ in physical and psychological risk (p = 0.142), unmet instrumental activities of daily living needs (p = 0.199), and provider shortcomings (p = 0.984).	6.5

NRCT = Non-randomized controlled trial; RCT = Randomized controlled trial.

The quality of studies of consumer directed care was the lowest of the three models examined. There were three randomized controlled trials [52-54], one non-randomized controlled trial [55] and two observational studies [56,57] that compared consumer-directed care to control groups [55-57]. It is notable that consumer directed care usually involves a budget for the purchase of services and usual care consumers may not have had received a similar value of services, such that any benefits may not have been due to the consumer involvement in directing care but the facilitation of easier access to services. Overall the results showed that consumer directed care improved satisfaction with care and community service use, but had little effect on clinical outcomes (see Table 1). Notably one study found that receiving consumer directed care may have increased psychological morbidity [54].

## Discussion

In summary, there was the most and highest quality evidence, including from randomized controlled trials, that case management improves clinical outcomes, decreases nursing home admission and hospital use. There was poorer quality evidence, mostly from non-randomized trials, that integrated care increases service use, and higher quality evidence from randomized trials that integrated care does not increase clinical outcomes. The lowest quality evidence was for consumer directed care, which appears to increase satisfaction with care and community service use but has little effect on consumer outcomes. Case management decreased use of services, possibly by decreasing the need for such services, but integrated care increased use of services, possibly by facilitating access to needed services.

These findings suggest that different models of home and community care have differing outcomes depending on their focus - case management focuses on consumer care, integrated care on an efficient system and consumer directed care on giving control to the consumer. Administrators and providers of services need to be explicitly clear as to the focus of their service and prioritization of outcomes. Improvement or maintenance of physical and mental health and functioning may be more important than delaying mortality, or improving satisfaction with services. An ideal model could incorporate multiple key elements - a fully integrated care system which facilitates access to health and community services, in which consumers receive case management to maximize clinical outcomes and prevent unnecessary institutionalization and hospital use, and where consumers have as much control of their own care as they wish.

The inconsistencies in results between studies are notable - the studies reviewed here were heterogeneous in their inclusion criteria, design, sample and methods of delivery. There was variability not just in the choice of instruments to measure outcomes, but the outcomes that were measured - these were based on the aim of each program. Most importantly, the health and social care systems in which the evaluations were conducted differ significantly - for instance the UK, Canada and Australia offer universal health and social care, whereas in the USA the majority of care is provided by insurance companies also known as health maintenance organizations. Successful programs would need to be skillfully adapted for other settings. That said, the patterns observed in these results are consistent with previous reviews of the individual models of care [9,12] suggesting that common lessons can be drawn from these studies despite their dissimilarities.

There are several limitations to this review. We did not attempt to search the grey literature, and thus could have missed service evaluations. We did not consider the cost-benefits of different models of community care. The divisions were not always clear between home and community care and other services such as primary health care and rehabilitation, requiring us to make subjective decisions on the inclusion of studies. There were overlaps between the different models of care. Integrated care models usually included case management, and consumer-directed care usually included the assessment and individualized care plan components of case management. One of the consumer-care trials explicitly attempted to increase integration [54]. We were not able to examine differences in the effects of community care between subgroups such as between participants with and without caregivers, or between participants with physical disabilities or cognitive impairment, or both. We only identified one paper of restorative home care [58] and could not include this model in the review. The evidence for restorative home care should be re-examined as further research is published [59].

A systematic review of randomized controlled trials provides the highest quality evidence of the efficacy of an intervention [60]. The second highest quality evidence is from randomized controlled trials where researchers can be confident that the intervention, not underlying differences between groups is the cause of different outcomes between groups. Examination of the studies included in this review reveal the difficulty in conducting randomized trials of care models that involve changes in care practices or whole care systems. A clustered randomized trial would be the best design to evaluate a model of care, however particularly for fully integrated care there would be substantial logistical barriers and high costs involved in such a study. Future evaluations of community and home care should give detailed descriptors of the service context, intervention and care received by controls, and should measure a broad range of outcomes clinical and service outcomes.

## Conclusions

This is the first systematic review comparing different models of non-medical home and community services for older persons. Each model impacts on different outcomes which relate to the focus of the model. Instead of asking which model is the best at improving outcomes, we should be asking how to combine the successful features of all three models to maximize outcomes.

## Competing interests

The authors declare that they have no competing interests.

## Authors' contributions

LFL and HB conceptualized the review. LFL and MY conducted the search and data compilation. All authors contributed to writing the paper and interpretation, and read and approved the final manuscript.

## Pre-publication history

The pre-publication history for this paper can be accessed here:

http://www.biomedcentral.com/1472-6963/11/93/prepub
